# Implications for sustaining secondary prophylaxis for rheumatic heart disease in Ethiopia: Uptake and determinants in a multicentre cross-sectional study

**DOI:** 10.1371/journal.pntd.0014703

**Published:** 2026-09-11

**Authors:** Eshetie Melese Birru, Zemikael Melese Birru, Yaschilal Muche Belayneh, Muluken Adela Alemu, Jane Oliver, Zemene Tigabu Kebede

**Affiliations:** 1 Medical School, Curtin University, Bentley, Australia; 2 Wesfarmers Centre for Vaccines and Infectious Diseases, The Kids Research Institute Australia, Nedlands, Australia; 3 UWA Centre for Child Health Research, The University of Western Australia, Nedlands, Australia; 4 Department of Pharmacology, College of Medicine and Health Sciences, University of Gondar, Gondar, Ethiopia; 5 Medical Teams International, Gondar, Ethiopia; 6 Department of Pharmacy, College of Medicine and Health Sciences, Wollo University, Dessie, Ethiopia; 7 Department of Pharmacy, College of Medicine and Health Sciences, Debre Tabor University, Debre Tabor, Ethiopia; 8 Department of Infectious Diseases, The Peter Doherty Institute for Infection and Immunity, The University of Melbourne, Parkville, Australia; 9 Department of Paediatrics, College of Medicine and Health Sciences, University of Gondar, Gondar, Ethiopia; Fukuoka University Hospital: Fukuoka Daigaku Byoin, JAPAN

## Abstract

**Background:**

Rheumatic heart disease (RHD) remains a major public health problem in low- and middle-income countries. Long-term benzathine penicillin G (BPG) secondary prophylaxis prevents recurrent acute rheumatic fever (ARF) and progressive valve damage, but maintaining adequate uptake (i.e., ≥80% dose) remains challenging in high-burden settings. We assessed BPG uptake, factors associated with high uptake, and its association with reported recurrent ARF manifestations among people with RHD in Ethiopia.

**Methods:**

A multicentre facility-based cross-sectional study was conducted among 400 participants with echocardiography-confirmed RHD attending three referral hospitals in Ethiopia between November 2023 and November 2024. Demographic, socioeconomic, geographic, healthcare-service and treatment-related factors associated with high uptake were evaluated using logistic regression (R version 4.6.1) with exposure-specific adjustment sets selected according to plausible confounding relationships. Adjusted odds ratios (aORs) with 95% confidence intervals (CIs) were reported.

**Results:**

Overall, 305/400 participants (76.3%) achieved ≥80% BPG uptake. The odds of high uptake decreased with increasing age (aOR per 10-year increase 0.80, 95% CI 0.69–0.94) and were higher among males than females (aOR 2.64, 95% CI 1.62–4.42). Living >50 km from an RHD follow-up facility was strongly associated with lower uptake compared with living <30 km away (aOR 0.10, 95% CI 0.04–0.22). Rural (aOR 0.28, 95% CI 0.14–0.52) and suburban residence (aOR 0.42, 95% CI 0.19–0.93) were also associated with lower uptake than urban residence. Compared with 12–24 months of prophylaxis, progressively lower uptake was observed with longer treatment duration (2–3 years: aOR 0.26, 95% CI 0.11–0.56; 3–4 years: aOR 0.26, 95% CI 0.10–0.61; > 4 years: aOR 0.07, 95% CI 0.03–0.15). Frequent injection pain was associated with lower uptake (aOR 0.32, 95% CI 0.17–0.59), whereas family support was associated with higher uptake (aOR 2.89, 95% CI 1.24–6.71). BPG uptake ≥80% was associated with substantially lower odds of reported recurrent ARF manifestations (aOR 0.25, 95% CI 0.14–0.44).

**Conclusions:**

Approximately three-quarters of participants achieved ≥80% BPG uptake. Geographic access, longer prophylaxis duration, injection pain, and limited family support were important correlates of lower uptake. Decentralised BPG delivery, improved injection acceptability, and sustained patient and family support may increase long-term secondary prophylaxis uptake in Ethiopia.

## Author summary

Rheumatic heart disease (RHD) remains an important cause of preventable illness in Ethiopia. Regular benzathine penicillin G (BPG) injections can prevent recurrent acute rheumatic fever and further heart-valve damage, but maintaining injections over many years can be challenging. We studied 400 people with RHD attending three referral hospitals in Ethiopia to determine how consistently they received prescribed BPG injections and identify factors associated with higher uptake. Approximately three-quarters received at least 80% of their prescribed injections during the preceding year. Lower uptake was particularly associated with living farther from the follow-up facility, living in non-urban areas, longer duration of prophylaxis, and frequent injection pain, while family support was associated with higher uptake. Participants with higher BPG uptake reported fewer recurrent acute rheumatic fever manifestations during the preceding year, although the study design cannot establish that higher uptake caused this difference. These findings suggest that maintaining long-term BPG prophylaxis depends on both healthcare accessibility and the cumulative burden of treatment. Improving access to safe BPG delivery, injection acceptability, reliable BPG supply, longitudinal follow-up, and sustained patient and family support may strengthen RHD control in Ethiopia.

## Introduction

Rheumatic heart disease (RHD), a chronic sequela of acute rheumatic fever (ARF), remains a significant global health burden, disproportionately affecting low- and middle-income countries (LMICs) where prevention and early intervention services remain limited [1–3]. ARF is an autoimmune reaction triggered in response to untreated pharyngitis and possibly skin infections caused by *Streptococcus pyogenes.* Progression to RHD results in permanent cardiac valve damage, which can lead to progressive heart failure, disability, and premature mortality [4,5].

RHD affects over 50 million people globally and accounts for more than 300,000 deaths annually. Approximately 23% of RHD cases reside in sub-Saharan Africa, which is disproportionally affected by this disease of poverty [6]. Ethiopia is among the highest-burden countries, with an estimated RHD population prevalence of 3.2%, one of the highest reported worldwide [7].

Secondary prophylaxis with intramuscular (IM) benzathine penicillin G (BPG), administered by trained clinical staff every 3–4 weeks, remains the cornerstone of RHD control. Maintaining adequate circulating penicillin concentrations prevents *S. pyogenes* exposure from triggering recurrent episodes of ARF, thereby preventing progression of cardiac damage. The World Health Organization (WHO) recommends that individuals with diagnosed ARF or RHD receive long-term secondary prophylaxis; either from the time of diagnosis until age 21 years, or for ten years after the last ARF episode, whichever period is longer [8]. Systematic reviews demonstrate that uptake of ≥80% of the recommended BPG injections per year reduces ARF recurrences by up to 96%; in contrast, uptake of <80% doses is associated with a four-fold increase in recurrence risk and an almost two-fold increase in mortality within six years [9,10].

Despite its proven effectiveness, uptake of BPG secondary prophylaxis remains suboptimal in many LMICs, with reported poor uptake ranging from 13% to 37% [11–13]. Barriers are multifactorial and include limited geographic access to health facilities where BPG can be administered, frequent BPG shortages, health providers being hesitant to administer BPG due to concerns about adverse reactions, and limited patient education [14–16]. Pain related to the administration of regular deep IM BPG injections is a well-documented barrier to long-term uptake [17,18]. However, evidence suggests that adequate uptake of BPG is achievable when culturally competent care, strong patient–provider relationships, and patient register-based follow-up systems are in place [10,19].

Previous Ethiopian studies have provided important evidence on BPG uptake and qualitative descriptions of patient-, provider-, and health-system barriers [16,20]. However, quantitative evidence simultaneously examining demographic and household circumstances, geographic access, healthcare-service factors, treatment duration, injection experience, and family support across multiple RHD follow-up centres remains limited.

Identifying independent determinants of successful uptake is essential for designing effective national RHD control strategies, prioritising interventions, and informing implementation of the WHO recommendations. Such evidence is particularly important in Ethiopia, where a national strategy to prevent, control, and treat RHD has not yet been established, and access to decentralised prophylaxis services remains limited, resulting in fragmented patient follow-up and inequitable access to long-term care. Therefore, this multicentre cross-sectional study aimed to identify independent patient-, socioeconomic-, clinical-, and health-system-related determinants associated with high uptake of BPG secondary prophylaxis among individuals living with RHD in Ethiopia.

## Methods

### Ethical statement

The study protocol was approved by the Institutional Review Board of the University of Gondar (VP/RTT/05/736/2023). Participants aged <18 years were enrolled following written informed consent from a parent or legal guardian, with child assent obtained for those aged >12 years, as applicable. Hospital administrations were notified through formal letters issued by the Amhara Public Health Institute.

### Study design and setting

A facility-based cross-sectional study was conducted between November 2023 and November 2024 among patients attending routine cardiac follow-up clinics. Data were collected prospectively at the time of clinic attendance from participants who had been prescribed secondary prophylaxis at three Ethiopian hospitals: Felge-Hiwot Referral Hospital in Bahir Dar, Dessie Referral Hospital in Dessie, and Debre Tabor Referral Hospital in Debre-Tabor in the Amhara region. Each hospital serves millions of people within its catchment area [21] and was the sole provider of RHD follow-up services in its respective area. Research data were collected using a piloted interviewer-administered semi-structured questionnaire, supplemented by clinical data extracted from medical records. This manuscript is presented according to the Modified STROBE guidelines [22].

### Study participants

Participants were eligible for recruitment with informed consent if they had echocardiography-confirmed RHD and had been prescribed IM BPG secondary prophylaxis; had received prophylaxis for at least 12 months before enrolment; and attended one of the participating outpatient clinics. Eligible participants were recruited consecutively during routine outpatient clinic visits until the target sample size was reached. Consecutive recruitment was used to minimise investigator selection bias by inviting all eligible patients. Participants under 18 years were enrolled with caregiver consent and child assent where relevant (age > 12 years old) [9].

### Data collection

Data were collected during routine cardiac outpatient visits using a semi-structured questionnaire administered by trained data collectors in the participant’s preferred local language. All questions were read aloud to facilitate consistent administration and participation irrespective of literacy level. Before recruitment, the questionnaire was first piloted among 25 RHD patients, with modifications subsequently made to the style and type of questions to enhance participant clarity and cultural appropriateness. The questionnaire was informed by previous literature [14,15] and collected information on i) sociodemographic data: age, sex, education, occupation, family income, household size, number of rooms; ii) healthcare access and service-related factors, including distance to the follow-up facility, BPG availability, health insurance, waiting time, and treatment- and transport-related costs iii) BPG secondary prophylaxis, including prescribed injection frequency, uptake during the preceding 12 months, injection pain and adverse effects; and (iv) perceived barriers and facilitators to maintaining secondary prophylaxis. Participants reporting missed injections were asked to identify the principal reason and any additional contributing barriers. The questionnaire also assessed recurrent ARF manifestations (e.g., carditis) during the preceding 12 months

Medical records were reviewed to verify BPG prophylaxis history and uptake and to obtain relevant clinical information, including echocardiographic findings, documented recurrent ARF manifestations, and physician notes. Where participant-reported information differed from the available clinical documentation, additional information from the medical records was reviewed, and used as the primary source for the relevant variable.

### Outcome measurement

The primary outcome was high uptake of secondary prophylaxis, defined as receiving ≥80% of doses in the previous 12 months, consistent with WHO recommendations [23]. The term “uptake” was used rather than adherence because receipt of scheduled BPG injections depends not only on individual behaviour but also on treatment availability, health-system accessibility, provider capacity, and continuity of care. The secondary outcome was participant-reported and/or recorded occurrence of one or more episodes of recurrent ARF manifestations (e.g., carditis) during the preceding 12 months. This outcome was analysed as a binary variable (yes/no).

### Sample size determination

Prior Ethiopian studies reported that approximately 35% of patients had poor secondary prophylaxis uptake [13,24,25]. Based on this expected proportion, the sample size required to estimate the primary outcome was calculated using a 95% confidence level (CI), a 5% margin of error, and a 10% allowance for non-response, resulting in a required sample of 389 participants. To ensure adequate power for analyses of factors associated with uptake, an additional sample size calculation was performed using the double population proportion formula in Epi Info, assuming a 95% CI, 80% power, and 1:1 exposure ratio. On this basis, the final sample size was set at 400 participants.

### Data analysis

Analyses were conducted using R version 4.6.1 (R Foundation for Statistical Computing, Vienna, Austria). Continuous variables were summarised using means and standard deviations, and categorical variables using frequencies and percentages. High BPG secondary prophylaxis uptake, defined as receiving ≥80% of prescribed injections in the preceding 12 months, was the primary outcome.

Associations with high BPG uptake were evaluated using binary logistic regression. Crude odds ratios (ORs) and 95% CIs were estimated, followed by adjusted analyses using exposure-specific models. Covariates were selected a priori based on plausible confounding relationships rather than automated stepwise procedures. Age and sex were included as core demographic covariates where appropriate, with additional socioeconomic, household, geographic, or treatment-related variables included when considered plausible confounders (S1 Table). Age was modelled continuously and expressed per 10-year increase. Its functional form was assessed by comparing linear and natural-spline specifications; there was no evidence that the spline improved model fit (likelihood-ratio test [LRT] p = 0.704), supporting use of a linear term.

For multi-category exposures, overall associations were evaluated using LRTs comparing models with and without the exposure while retaining the same adjustment set. Category-specific adjusted ORs (aORs) and 95% CIs were interpreted alongside the corresponding overall exposure-level tests. Model diagnostics included assessment of multicollinearity, separation, influential observations, discrimination, and calibration. No problematic multicollinearity or complete or quasi-complete separation was identified in the principal models. Calibration was assessed graphically by comparing observed and model-predicted probabilities. Sensitivity analyses excluding influential observations were conducted to assess the robustness of the principal estimates.

The secondary outcome was the occurrence of one or more recurrent ARF during the preceding 12 months. The association between BPG uptake and reported recurrent ARF manifestations was evaluated using logistic regression, adjusting for age, sex, and duration of BPG secondary prophylaxis. Model discrimination, calibration and influence were assessed, and sensitivity analyses examined the robustness of the association. All statistical tests were two-sided. Interpretation emphasised effect estimates, 95% CIs, and overall exposure-level tests rather than statistical significance alone.

## Results

### Participant characteristics

Of 553 patients screened, 400 were enrolled and included in the analysis. Of the 153 individuals not enrolled, 61 did not meet the eligibility criteria, 72 declined participation, and 20 had insufficient clinical records to confirm eligibility/or ascertain the primary outcome of BPG uptake during the preceding 12 months. No participants were excluded from the analysis after enrolment, and there were no missing values among the final analytical variables (n = 400). The mean age of enrolled participants (n = 400) was 26.5 years (SD ± 15.1), and most were female (53.3%). Over half of the participants (51.5%) resided in rural areas. Approximately one-third (32.5%) had no formal education, while 18% had completed college- or university-level education. The majority (71.5%) were covered by community-based health insurance. Nearly 30% of households reported a monthly income <2,000 Ethiopian Birr, whereas 10% reported an income >8,000 Birr. Additionally, 22.5% of participants lived in single-room dwellings, and half (50%) resided in households with five or more members (Table 1).

**Table 1:** Sociodemographic characteristics of the 400 study participants according to BPG secondary prophylaxis uptake, Ethiopia, 2023–2024.

Characteristics		N (%)
Age (years), mean ± SD		26.5 ± 15.1
Sex	Male	187 (46.7)
	Female	213 (53.3)
Highest educational status (household)	No formal education	130 (32.5)
	Primary school	106 (26.5)
	Secondary school	92 (23.0)
	College/university graduate	72 (18.0)
Residential area	Rural	206 (51.5)
	Suburban	121 (30.3)
	Urban	73 (18.3)
Family income (average monthly income, Ethiopian Birr)	<2,000	119 (29.8)
	2,000-4,999	142 (35.5)
	5,000-7,999	98 (24.5)
	>8,000	41 (10.3)
Health insurance cover	Yes	286 (71.5)
	No	114 (28.5)

### Factors associated with high BPG uptake

As detailed in Table 2, increasing age was associated with lower BPG uptake. After adjustment for sex (S1 Table), each 10-year increase in age was associated with approximately 20% lower odds of achieving ≥80% uptake (aOR 0.80, 95% CI 0.69–0.94; p = 0.005). Males had higher adjusted odds of high uptake than females (aOR 2.64, 95% CI 1.62–4.42; p < 0.001). Household educational level was not associated with uptake overall after adjustment (LRT p = 0.213). Similarly, household income (p = 0.144) and household size (p = 0.138) showed no clear overall adjusted associations. The number of rooms in the household was associated with uptake overall (LRT p = 0.010). Compared with participants living in single-room households, those living in households with two rooms had greater odds of high uptake (aOR 2.42, 95% CI 1.33–4.47), as did those living in households with three rooms (aOR 3.10, 95% CI 1.39–7.24).

**Table 2:** Factors associated with high BPG secondary prophylaxis uptake, Ethiopia 2023/24.

Exposure	Category/comparison	<80% uptake (n=95) n (%)	≥80% uptake (n=305) n (%)	Crude OR (95% CI)	Adjusted OR (95% CI)	Overall P value
Age, years, mean ± SD	Per 10-year increase	30.3 ± 16.3	25.3 ± 14.5	0.81 (0.70–0.94)	**0.80 (0.69–0.94)**	0.005
Sex	Female	67 (70.5%)	146 (47.9%)	-	-	<0.001
	Male	28 (29.5%)	159 (52.1%)	2.61 (1.60–4.33)	**2.64 (1.62–4.42)**	
Highest household educational level	No formal education	39 (41.1%)	91 (29.8%)	-	-	0.213
	Primary school	32 (33.7%)	74 (24.3%)	0.99 (0.57–1.74)	1.21 (0.66–2.26)	
	Secondary school	17 (17.9%)	75 (24.6%)	1.89 (1.00–3.68)	1.62 (0.78–3.41)	
	College/university	7 (7.4%)	65 (21.3%)	3.98 (1.77–10.22)	**2.75 (1.03–8.03)**	
Monthly household income (ETB)	<2,000	42 (44.2%)	77 (25.2%)	-	-	0.144
	2,000–4,999	32 (33.7%)	110 (36.1%)	1.87 (1.09–3.25)	1.83 (0.99–3.41)	
	5,000–7,999	15 (15.8%)	83 (27.2%)	3.02 (1.58–6.03)	2.23 (0.98–5.22)	
	≥8,000	6 (6.3%)	35 (11.5%)	3.18 (1.31–8.95)	1.98 (0.74–6.03)	
Household size	3	21 (22.1%)	90 (29.5%)	-	-	0.138
	4	13 (13.7%)	74 (24.3%)	1.33 (0.63–2.89)	1.55 (0.71–3.50)	
	≥5	61 (64.2%)	141 (46.2%)	0.54 (0.30–0.93)	0.75 (0.39–1.40)	
Number of rooms in household	1	38 (40.0%)	52 (17.0%)	-	-	0.010
	2	39 (41.1%)	135 (44.3%)	2.53 (1.46–4.40)	**2.42 (1.33–4.47)**	
	3	12 (12.6%)	73 (23.9%)	4.45 (2.17–9.64)	**3.10 (1.39–7.24)**	
	≥4	6 (6.3%)	45 (14.8%)	5.48 (2.26–15.49)	**2.79 (1.03–8.50)**	
Residential area	Urban	15 (15.8%)	106 (34.8%)	-	-	<0.001
	Rural	64 (67.4%)	142 (46.6%)	0.31 (0.16–0.57)	**0.28 (0.14–0.52)**	
	Suburban	16 (16.8%)	57 (18.7%)	0.50 (0.23–1.10)	**0.42 (0.19–0.93)**	
Distance to RHD follow-up facility	<30 km	54 (56.8%)	228 (74.8%)	-	-	<0.001
	30–50 km	16 (16.8%)	65 (21.3%)	0.96 (0.53–1.84)	0.79 (0.42–1.55)	
	>50 km	25 (26.3%)	12 (3.9%)	0.11 (0.05–0.24)	**0.10 (0.04–0.22)**	
Health insurance coverage	No	21 (22.1%)	93 (30.5%)	-	-	0.351
	Yes	74 (77.9%)	212 (69.5%)	0.65 (0.37–1.10)	1.38 (0.70–2.72)	
Waiting time for hospital services	<2 hours	16 (16.8%)	77 (25.2%)	-	-	<0.001
	2–3 hours	27 (28.4%)	140 (45.9%)	1.08 (0.54–2.10)	2.09 (0.97–4.46)	
	>3 hours	52 (54.7%)	88 (28.9%)	0.35 (0.18–0.65)	**0.56 (0.27–1.13)**	
Comorbidity	No	81 (85.3%)	272 (89.2%)	-	-	0.469
	Yes	14 (14.7%)	33 (10.8%)	0.70 (0.37–1.41)	0.77 (0.39–1.59)	
Duration of BPG secondary prophylaxis	1–2 years	10 (10.5%)	139 (45.6%)	-	-	<0.001
	2–3 years	24 (25.3%)	79 (25.9%)	0.24 (0.10–0.51)	**0.26 (0.11–0.56)**	
	3–4 years	14 (14.7%)	48 (15.7%)	0.25 (0.10–0.59)	**0.26 (0.10–0.61)**	
	>4 years	47 (49.5%)	39 (12.8%)	0.06 (0.03–0.12)	**0.07 (0.03–0.15)**	
Family support for healthcare*	No	16 (16.8%)	31 (10.2%)	-	-	0.013
	Yes	79 (83.2%)	274 (89.8%)	1.79 (0.91–3.40)	**2.89 (1.24–6.71)**	
Frequent BPG injection pain	No	19 (20.0%)	122 (40.0%)	-	-	<0.001
	Yes	76 (80.0%)	183 (60.0%)	0.38 (0.21–0.64)	**0.32 (0.17–0.59)**	

*Defined as any financial, transport, emotional, or medication-related support from family/caregivers. CI, confidence interval; ETB, Ethiopian Birr; OR, odds ratio; RHD, rheumatic heart disease.

Residential area was strongly associated with uptake (overall p < 0.001). Compared with urban residents, rural participants had lower odds of high uptake (aOR 0.28, 95% CI 0.14–0.52), as did suburban participants (aOR 0.42, 95% CI 0.19–0.93). Distance to the RHD follow-up facility showed one of the strongest associations. Participants living 30–50 km away had similar odds of high uptake to those living <30 km away (aOR 0.79, 95% CI 0.42–1.55). In contrast, participants living >50 km away had approximately 90% lower odds of achieving high uptake (aOR 0.10, 95% CI 0.04–0.22; overall p < 0.001).

Waiting time for hospital services was associated with uptake overall (LRT p < 0.001), although the relationship was non-linear (non-monotonic pattern across categories). Relative to waiting <2 hours, the aORs was 0.56 (95% CI 0.27–1.13) for waiting >3 hours. The individual category estimates were imprecise and should therefore not be interpreted as evidence of a simple dose-response relationship. Duration of BPG prophylaxis was strongly associated with uptake (overall p < 0.001). Compared with participants treated for 12–24 months, odds of high uptake were lower among those treated for 2–3 years (aOR 0.26, 95% CI 0.11–0.56), 3–4 years (aOR 0.26, 95% CI 0.10–0.61), and >4 years (aOR 0.07, 95% CI 0.03–0.15). Family support was associated with higher uptake (aOR 2.89, 95% CI 1.24–6.71; p = 0.013), whereas frequent BPG injection pain was associated with lower uptake after adjustment for age, sex and duration of prophylaxis (aOR 0.32, 95% CI 0.17–0.59; p < 0.001).

Model diagnostics identified no problematic multicollinearity or complete or quasi-complete separation. The principal distance model showed moderate discrimination (AUC 0.705, 95% CI 0.641–0.770), with broadly acceptable graphical calibration (S2 Table; S1 Fig). The principal associations were robust to additional adjustment and exclusion of influential observations; for example, the association for residence >50 km from the follow-up facility changed minimally after exclusion of the most influential observation (aOR 0.09, 95% CI 0.04–0.20) (S3 Table). There was evidence that the association between reported BPG side effects and high uptake varied according to distance from the follow-up facility (interaction LRT χ² = 8.94, df = 2, p = 0.011). Among participants living <30 km from the facility, reported side effects were associated with higher odds of high uptake (OR 2.98, 95% CI 1.15–7.73), whereas the direction of association differed among those living 30–50 km away (OR 0.25, 95% CI 0.06–1.01). Estimates in the more distant strata were imprecise; therefore, these findings were considered exploratory (S4 Table).

### Perceived primary barriers to BPG uptake

When asked to identify perceived primary barrier and/or reason for missed doses, participants most frequently cited the cost of follow-up care, including transport and service fees (30.3%). The second most common barrier was related to healthcare providers being reluctant to administering BPG (25.0%), largely driven by their own concern about anaphylaxis risk (Table 3).

**Table 3:** Barriers to high secondary prophylaxis uptake.

Barriers	n (%)
Cost of follow-up care	121 (30.3)
Healthcare providers’ reluctance to administer BPG	100 (25.0)
Lack of awareness about the disease	80 (20.0)
BPG shortages	18 (4.5)
Other (e.g., security, long waiting time)	33 (8.3)

### BPG uptake and recurrent ARF

Overall, 183/400 participants (45.8%) reported at least one episode of recurrent ARF manifestations during the preceding 12 months. There was a marked difference according to BPG uptake. Among participants with <80% uptake, 71/95 (74.7%) reported manifestations, compared with 112/305 (36.7%) among participants achieving ≥80% uptake. In unadjusted analysis, ≥ 80% uptake was associated with substantially lower odds of reported recurrent ARF attack than <80% uptake (OR 0.20, 95% CI 0.12–0.33; p < 0.001). The association remained after adjustment for age and sex (aOR 0.23, 95% CI 0.13–0.39; p < 0.001) and after further adjustment for duration of BPG prophylaxis (aOR 0.25, 95% CI 0.14–0.44; p < 0.001).

The duration-adjusted recurrent ARF manifestation model showed no evidence of problematic multicollinearity or separation and demonstrated moderate discrimination (AUC 0.718, 95% CI 0.667–0.768), with broadly acceptable graphical calibration (S5 Table; S2 Fig). Exclusion of the observation with the highest Cook's distance resulted in minimal change in the association between high uptake and reported recurrent ARF (aOR 0.24, 95% CI 0.13–0.41), indicating that the result was not driven by a single influential observation.

## Discussion

This multicentre study provides important insights into the determinants of uptake of BPG secondary prophylaxis among people prescribed secondary prophylaxis in Ethiopia—one of the countries with the highest documented global RHD burden [3,26]. Approximately three-quarters received ≥80% of prescribed BPG injections during the preceding year, which is higher than pooled estimates reported in two recent systematic reviews from LMIC settings (58.5% and 46%) (29,30). This proportion is within the range observed in two previous Ethiopian hospital-based studies [13,24], but must be interpreted in context. Our sample consisted entirely of patients attending outpatient follow-up appointments, which may have preferentially included individuals who remained engaged with care and therefore may overestimate uptake in the broader RHD population. Given the established effectiveness of regular BPG prophylaxis in preventing recurrent ARF [9,27], the finding that nearly one-quarter of participants did not meet this threshold, indicates they are at substantial risk of preventable ARF episodes and progressive valve damage.

The strongest associations with high uptake involved geographic access, duration of prophylaxis, injection-related pain, and family support. Older age and non-urban residence were also associated with lower uptake, while males had higher uptake than females. Collectively, these findings suggest that sustained prophylaxis reflects the cumulative accessibility and treatment burden of long-term care rather than patient behaviour alone.

In areas where RHD is prevalent, the WHO recommends register-based programs as a core component of RHD control to support delivery of secondary prophylaxis and longitudinal patient management [28]. National integrated prevention and registry-based programs have been widely advocated as an effective framework for improving clinical care, surveillance, and continuity of secondary prophylaxis in RHD populations [19,29]. Such a nationally coordinated programme is not currently established in Ethiopia despite the substantial burden of RHD reported. Registry-based RHD programs implemented in some African countries, including Uganda and Rwanda, have demonstrated improved case detection and retention in care following regional expansion of registry services [19,30]. Similarly, decentralized registry-supported RHD programs have shown feasibility in strengthening secondary prophylaxis delivery at primary healthcare level and guiding service planning in resource-limited settings [31]. A national register-based programme, particularly if integrated with decentralised BPG delivery, could facilitate systematic follow-up, recall after missed injections, identification of patients at risk of disengagement, and planning of BPG supply.

Several socioeconomic and household characteristics showed category-specific associations with uptake; however, the overall adjusted associations for household education, household income, and household size were not supported by exposure-level likelihood-ratio tests. These findings should therefore not be interpreted as evidence of independent socioeconomic effects. In contrast, the number of rooms in the household showed an overall association with uptake, although this measure is likely to represent a broader and imperfect proxy for household socioeconomic circumstances. The findings nevertheless reinforce the potential importance of structural conditions affecting patients’ capacity to maintain repeated healthcare attendance, while avoiding attribution of poor uptake solely to individual socioeconomic characteristics [14,15].

Geographic accessibility emerged as a critical factor associated with BPG uptake. There was little evidence of reduced uptake among those living 30–50 km away, suggesting that the greatest access disadvantage may occur at more extreme travel distances. In Ethiopia, where BPG administration is largely restricted to higher-level hospitals, distance may impose repeated burdens related to travel costs, time loss, and missed work opportunities. Embedding decentralised delivery pathways—such as outreach clinics, mobile teams, or integration into primary healthcare services—within a national registry infrastructure could reduce these barriers while preserving longitudinal patient tracking. Such models have demonstrated feasibility in comparable resource-limited settings and may provide a scalable mechanism to extend services to underserved rural populations [32,33].

Healthcare provider reluctance to administer BPG due to fear of anaphylaxis represents a potentially important health-system barrier identified in previous works in this country [16,20] and many other areas globally [34]. Although provider behaviour was not directly measured as an exposure in the present analysis, addressing provider-level barriers requires structured and sustained capacity-building strategies. Standardised training in safe BPG administration, recognition and management of anaphylaxis, appropriate injection technique, and pain mitigation could support expansion of BPG delivery beyond specialist facilities [34,35]..

These determinants are interdependent and operate across multiple levels of the health system, reinforcing the need for integrated rather than fragmented service-delivery models. Socioeconomic disadvantage, distance from healthcare facilities, provider hesitancy, and injection-related discomfort collectively represent interlinked barriers that contribute to declining adherence over time [14,15]. A nationally coordinated register-based secondary prophylaxis program with decentralised outreach services offers a practical mechanism to address these challenges simultaneously. By enabling systematic follow-up, recall of missed doses, monitoring of patient-level risk factors, and forecasting of BPG supply needs, such a program could transform fragmented service delivery into a proactive, patient-centred system. Importantly, registry-based systems would also allow early identification of patients at risk of long-term disengagement, enabling sustained counselling and supportive care to counteract injection fatigue and maintain lifelong adherence.

Injection-related burden was independently associated with uptake. Pain associated with repeated deep IM injections is a recognised barrier, particularly for children and adolescents. Studies in Australia and globally show that repeated painful injections can contribute to emotional distress, anticipatory anxiety, and long-term disengagement from care [32,33]. Interventions such as buffered lidocaine, evidence-based injection techniques, appropriate staff training, and other validated pain-reduction strategies should therefore form part of efforts to improve treatment acceptability. Alternative approaches to prolonged BPG delivery may also warrant investigation where their safety and effectiveness can be established [36].

Duration of prophylaxis showed one of the strongest associations with uptake. This pattern is consistent with increasing difficulty sustaining prophylaxis over time and may reflect cumulative challenges such as injection fatigue, declining motivation, and ongoing exposure to structural barriers [33,37]. Importantly, these finding highlight that the challenge in long-term secondary prophylaxis is not simply initiating treatment but maintaining patient engagement over the extended period. However, because duration of prophylaxis and uptake were assessed within a cross-sectional study, the observed gradient should not be interpreted as demonstrating that longer treatment duration itself causes declining uptake. Differences between patients at different stages of treatment and other unmeasured factors may contribute to the association. Early counselling at treatment initiation, combined with structured and sustained follow-up, may therefore be critical to supporting long-term adherence. A register-based secondary prophylaxis program could play a key role in maintaining engagement by enabling continuity of care, strengthening relationships between providers and patients and families, and facilitating regular contact to reinforce health literacy and sustained health service utilisation.

Family support was positively associated with prophylaxis uptake. Long-term injectable prophylaxis requires repeated attendance over many years, and family members may facilitate transport, appointment attendance, financial support, treatment reminders, and persistence with care. This finding suggests that family engagement should be considered alongside health-system interventions when designing strategies to sustain secondary prophylaxis. Uptake also varied according to age and sex. Each 10-year increase in age was associated with approximately 20% lower odds of high uptake (aOR 0.80, 95% CI 0.69–0.94), while males had higher odds of high uptake than females (aOR 2.64, 95% CI 1.62–4.42). The age association may partly reflect cumulative treatment burden or differences in engagement with long-term care, although these mechanisms were not directly assessed. Similarly, the observed sex difference may reflect differences in healthcare access, competing responsibilities, social support, or other contextual factors. These associations therefore warrant further investigation rather than causal interpretation.

Higher uptake in this study was strongly associated with markers of socioeconomic advantage, including higher educational attainment, household income, and housing conditions. These indicators likely reflect improved capacity to meet direct and indirect healthcare costs, which were identified by nearly one-third of participants as a major barrier to sustained prophylaxis attendance. Rather than representing isolated patient-level barriers, these findings highlight systemic inequities that limit continuity of care [14,15]. Within a national register-based secondary prophylaxis framework, such socioeconomic vulnerability could be systematically identified, enabling targeted support mechanisms such as transport subsidies, flexible scheduling, or community-based delivery, thereby reducing financial barriers that disproportionately affect disadvantaged households. High BPG uptake was also strongly associated with a lower frequency of reported recurrent ARF manifestations during the preceding 12 months. (≥80% uptake was associated with 75% lower odds of reported ARF attacks (aOR 0.25, 95% CI 0.14–0.44)). This association is consistent with the established role of regular secondary prophylaxis in preventing recurrent ARF and subsequent cardiac injury.

Taken together, the findings indicate that maintaining BPG secondary prophylaxis is shaped by interacting geographic, treatment-related, household, and health-system factors. A coordinated strategy combining decentralised BPG delivery with register-based longitudinal follow-up, reliable medicine supply, pain-reduction strategies, family engagement, and appropriately trained healthcare providers may therefore be more effective than interventions addressing individual patient behaviour alone. Such an approach could enable earlier identification of declining uptake and provide targeted support to patients facing substantial access or long-term treatment burdens. Prospective implementation studies are needed to determine whether these strategies improve sustained BPG uptake and clinical outcomes in Ethiopia [23,38]..

This study has several limitations. First, its cross-sectional design precludes causal inference. Second, although uptake information was supported by available clinical records, recall and measurement error remain possible. Third, the secondary outcome of recurrent ARF manifestations (e.g., carditis) was self-reported and was not independently clinically adjudicated; it should therefore not be interpreted as confirmed recurrent ARF or clinically confirmed rheumatic carditis. Fourth, the absence of provider-level perspectives and health system capacity data constrained the assessment of service-delivery factors. Fifth, selection bias may have occurred because participants were recruited from hospital-based follow-up clinics; individuals with very poor engagement, including those receiving few or no BPG injections were unlikely to be captured, and true population-level uptake may therefore be lower than reported. This selection process could also have influenced exposure–uptake associations if factors affecting clinic attendance were related to both the exposures examined and BPG uptake. In addition, 20 individuals screened for participation had insufficient clinical documentation to establish eligibility and/or ascertain BPG uptake and were therefore not enrolled; if these individuals differed systematically from included participants, some selection bias may have occurred. Finally, some exposures were based on participant report and residual confounding cannot be excluded despite the use of prespecified exposure-specific adjustment sets. Nevertheless, this multicentre study provides detailed quantitative evidence on secondary-prophylaxis uptake and associated factors across diverse hospital settings in Ethiopia. While generalisability beyond the study sites should be interpreted with appropriate caution, the inclusion of geographically and demographically varied catchment populations supports the relevance of the findings within similar public hospital contexts. The use of exposure-specific confounder adjustment, and sensitivity and model-diagnostic analyses further supports the robustness of the principal associations.

## Conclusion

Approximately three-quarters of participants attending RHD follow-up services achieved ≥80% BPG secondary prophylaxis uptake. Lower uptake was strongly associated with greater distance to care, longer duration of prophylaxis, non-urban residence, and frequent injection pain, while family support was associated with higher uptake. High uptake was also associated with substantially lower odds of reported recurrent ARF manifestations, although causal inference cannot be made. These findings highlight the cumulative access and treatment burden of maintaining long-term secondary prophylaxis and support integrated strategies combining decentralised BPG delivery with appropriate level of care, register-based longitudinal follow-up, improved injection acceptability, reliable BPG supply, and sustained patient and family support.

## Supporting information

S1 Data Minimal anonymized dataset underlying the results reported in this manuscript. (CSV)

S1 Table Exposure-specific adjustment sets and overall tests of association with high BPG secondary prophylaxis uptake. (DOCX)

S2 Table Diagnostic and sensitivity assessments of the logistic regression models for high BPG secondary prophylaxis uptake. (DOCX)

S3 Table Sensitivity analyses assessing the robustness of principal associations with high BPG secondary prophylaxis uptake. (DOCX)

S4 Table Model-adjusted association between reported BPG side effects and high BPG secondary prophylaxis uptake according to distance from the RHD follow-up facility. (DOCX)

S5 Table Sensitivity and model-diagnostic analyses for the secondary outcome of reported recurrent ARF manifestations. (DOCX)

S1 Checklist STROBE Implications for sustaining secondary prophylaxis for rheumatic heart disease in Ethiopia: Uptake and determinants in a multicentre cross-sectional study. (DOCX)

S1 Fig Calibration plot for the adjusted distance–BPG uptake model. (DOCX)

S2 Fig Calibration plot for the secondary-outcome model of reported recurrent ARF manifestations. (DOCX)
