# Supplementary material for: Implications for sustaining secondary prophylaxis for rheumatic heart disease in Ethiopia: Uptake and determinants in a multicentre cross-sectional study
Source: PLoS Negl Trop Dis. 2026 Sep 11;20(9):e0014703. doi: 10.1371/journal.pntd.0014703 (PMC13588498; doi:10.1371/journal.pntd.0014703)
Supplement: Supplementary file 1 [file pntd.0014703.s002.docx]

**S1Table. Exposure-specific adjustment sets and overall tests of association with high BPG secondary prophylaxis uptake**

| Exposure | Prespecified adjustment set | LRT χ² | df | Overall P value |
| --- | --- | --- | --- | --- |
| Age, per 10-year increase | Sex | — | 1 | 0.005 |
| Sex | Age | — | 1 | <0.001 |
| Highest household educational level | Age, sex, residential area | 4.49 | 3 | 0.213 |
| Monthly household income | Age, sex, household educational level, residential area | 5.41 | 3 | 0.144 |
| Household size | Age, sex, residential area | 3.97 | 2 | 0.138 |
| Number of rooms in household | Age, sex, household income, household size, residential area | 11.40 | 3 | 0.010 |
| Residential area | Age, sex | 17.53 | 2 | <0.001 |
| Distance to RHD follow-up facility | Age, sex | 34.50 | 2 | <0.001 |
| Health insurance coverage | Age, sex, household income, household educational level, residential area | — | 1 | 0.351 |
| Waiting time for hospital services | Age, sex, residential area | 20.76 | 2 | <0.001 |
| Comorbidity | Age, sex | 0.51 | 1 | 0.475 |
| Duration of BPG secondary prophylaxis | Age, sex | 51.43 | 3 | <0.001 |
| Family support for healthcare | Age, sex, household size | — | 1 | 0.013 |
| Frequent BPG injection pain | Age, sex, duration of BPG prophylaxis | — | 1 | <0.001 |

ORs and 95% CIs were estimated using binary logistic regression. Adjusted estimates were derived from exposure-specific models with covariates selected a priori according to plausible confounding relationships. Age was modelled per 10-year increase. Overall P values for multi-category exposures were obtained using likelihood-ratio tests. BPG, benzathine penicillin G; CI, confidence interval; ETB, Ethiopian birr; RHD, rheumatic heart disease.
