# Supplementary material for: Implications for sustaining secondary prophylaxis for rheumatic heart disease in Ethiopia: Uptake and determinants in a multicentre cross-sectional study
Source: PLoS Negl Trop Dis. 2026 Sep 11;20(9):e0014703. doi: 10.1371/journal.pntd.0014703 (PMC13588498; doi:10.1371/journal.pntd.0014703)
Supplement: Supplementary file 2 [file pntd.0014703.s003.docx]

**S2 Table. Diagnostic and sensitivity assessments of the logistic regression models**

| Diagnostic | Result | Interpretation |
| --- | --- | --- |
| Missing data in final analytical variables | None | Complete data for variables included in the analyses |
| Age linear vs spline | LRT p=0.704 | No evidence supporting nonlinear specification |
| Age model AIC | Linear 434.9; spline 438.1 | Linear specification preferred |
| Separation, principal distance model | None detected | Finite maximum-likelihood estimates |
| Distance model AUC | 0.705 (95% CI 0.641–0.770) | Moderate discrimination |
| Maximum Cook's distance, distance model | 0.020 | No single highly influential observation |
| Maximum leverage, distance model | 0.043 | No extreme leverage |
| Distance >50 km after excluding most influential observation | aOR 0.09 (95% CI 0.04–0.20) | Association remained stable |
| Separation, common sensitivity model | None detected | No evidence of separation |
| Common model convergence | Converged | Model was numerically estimable |
| Side-effects × distance interaction | LRT p=0.011 | Evidence of interaction; interpreted exploratorily |
| Calibration | Observed versus predicted probabilities assessed graphically | See Supplementary Figure S1 |

LRT, likelihood-ratio test; AIC, Akaike Information Criterion
