# Supplementary material for: Implications for sustaining secondary prophylaxis for rheumatic heart disease in Ethiopia: Uptake and determinants in a multicentre cross-sectional study
Source: PLoS Negl Trop Dis. 2026 Sep 11;20(9):e0014703. doi: 10.1371/journal.pntd.0014703 (PMC13588498; doi:10.1371/journal.pntd.0014703)
Supplement: Supplementary file 3 [file pntd.0014703.s004.docx]

**S3 Table. Sensitivity analyses assessing robustness of principal associations with high BPG secondary prophylaxis uptake**

| Exposure/comparison | Primary model aOR (95% CI) | Sensitivity analysis | Sensitivity aOR (95% CI) |
| --- | --- | --- | --- |
| Distance: 30–50 vs <30 km | 0.79 (0.42–1.55) | Additional adjustment for residence | 0.82 (0.43–1.61) |
| Distance: >50 vs <30 km | 0.10 (0.04–0.22) | Additional adjustment for residence | 0.12 (0.05–0.26) |
| Distance: 30–50 vs <30 km | 0.79 (0.42–1.55) | Excluding observation with highest Cook's distance | 0.79 (0.42–1.54) |
| Distance: >50 vs <30 km | 0.10 (0.04–0.22) | Excluding observation with highest Cook's distance | 0.09 (0.04–0.20) |
| BPG duration: 2–3 vs 12–24 months | 0.26 (0.11–0.56) | Excluding most influential observation | 0.24 (0.10–0.52) |
| BPG duration: 3–4 vs 12–24 months | 0.26 (0.10–0.61) | Excluding most influential observation | 0.23 (0.09–0.57) |
| BPG duration: >4 years vs 12–24 months | 0.07 (0.03–0.15) | Excluding most influential observation | 0.06 (0.03–0.14) |
| Frequent injection pain: Yes vs No | 0.32 (0.17–0.59) | Excluding most influential observation | 0.29 (0.15–0.55) |

Sensitivity analyses were performed to evaluate whether principal associations were materially affected by additional adjustment or individual influential observations. Estimates were essentially unchanged, supporting robustness of the principal associations. aOR, adjusted odds ratio; BPG, benzathine penicillin G; CI, confidence interval.
