# Supplementary material for: Implications for sustaining secondary prophylaxis for rheumatic heart disease in Ethiopia: Uptake and determinants in a multicentre cross-sectional study
Source: PLoS Negl Trop Dis. 2026 Sep 11;20(9):e0014703. doi: 10.1371/journal.pntd.0014703 (PMC13588498; doi:10.1371/journal.pntd.0014703)
Supplement: Supplementary file 4 [file pntd.0014703.s005.docx]

**S4 Table. Model-adjusted association between reported BPG side effects and high uptake according to distance from the RHD follow-up facility**

| Distance to facility | Adjusted probability of ≥80% uptake: no side effects | Adjusted probability: side effects | Side effects vs no side effects OR (95% CI) | P value |
| --- | --- | --- | --- | --- |
| <30 km | 86.2% (80.1–90.7%) | 94.9% (87.6–98.0%) | 2.98 (1.15–7.73) | 0.025 |
| 30–50 km | 87.0% (75.0–93.8%) | 62.9% (34.4–84.5%) | 0.25 (0.06–1.01) | 0.052 |
| >50 km | 29.4% (7.8–67.2%) | 34.5% (15.1–61.0%) | 1.26 (0.18–8.86) | 0.814 |

**Overall side-effects × distance interaction:** LRT χ²=8.94, df=2, **p=0.011.** Probabilities and contrasts were derived from the interaction model containing reported BPG side effects, distance to the follow-up facility, age, sex, duration of BPG prophylaxis and injection pain. Model-based probabilities were averaged over sex, prophylaxis duration and injection-pain categories, with continuous covariates evaluated according to the estimated marginal-means reference grid. Because the interaction analysis was exploratory and estimates in the more distant strata were imprecise, subgroup estimates should be interpreted cautiously.
