# Supplementary material for: Implications for sustaining secondary prophylaxis for rheumatic heart disease in Ethiopia: Uptake and determinants in a multicentre cross-sectional study
Source: PLoS Negl Trop Dis. 2026 Sep 11;20(9):e0014703. doi: 10.1371/journal.pntd.0014703 (PMC13588498; doi:10.1371/journal.pntd.0014703)
Supplement: Supplementary file 5 [file pntd.0014703.s006.docx]

**Supplementary Table S3. Sensitivity analyses assessing robustness of principal associations with high BPG secondary prophylaxis uptake**

| Exposure/comparison | Primary model aOR (95% CI) | Sensitivity analysis | Sensitivity aOR (95% CI) |
| --- | --- | --- | --- |
| Distance: 30–50 vs <30 km | 0.79 (0.42–1.55) | Additional adjustment for residence | 0.82 (0.43–1.61) |
| Distance: >50 vs <30 km | 0.10 (0.04–0.22) | Additional adjustment for residence | 0.12 (0.05–0.26) |
| Distance: 30–50 vs <30 km | 0.79 (0.42–1.55) | Excluding observation with highest Cook's distance | 0.79 (0.42–1.54) |
| Distance: >50 vs <30 km | 0.10 (0.04–0.22) | Excluding observation with highest Cook's distance | 0.09 (0.04–0.20) |
| BPG duration: 2–3 vs 12–24 months | 0.26 (0.11–0.56) | Excluding most influential observation | 0.24 (0.10–0.52) |
| BPG duration: 3–4 vs 12–24 months | 0.26 (0.10–0.61) | Excluding most influential observation | 0.23 (0.09–0.57) |
| BPG duration: >4 years vs 12–24 months | 0.07 (0.03–0.15) | Excluding most influential observation | 0.06 (0.03–0.14) |
| Frequent injection pain: Yes vs No | 0.32 (0.17–0.59) | Excluding most influential observation | 0.29 (0.15–0.55) |

Sensitivity analyses were performed to evaluate whether principal associations were materially affected by additional adjustment or individual influential observations. Estimates were essentially unchanged, supporting robustness of the principal associations. aOR, adjusted odds ratio; BPG, benzathine penicillin G; CI, confidence interval.

**Supplementary Table S4. Model-adjusted association between reported BPG side effects and high uptake according to distance from the RHD follow-up facility**

| Distance to facility | Adjusted probability of ≥80% uptake: no side effects | Adjusted probability: side effects | Side effects vs no side effects OR (95% CI) | P value |
| --- | --- | --- | --- | --- |
| <30 km | 86.2% (80.1–90.7%) | 94.9% (87.6–98.0%) | 2.98 (1.15–7.73) | 0.025 |
| 30–50 km | 87.0% (75.0–93.8%) | 62.9% (34.4–84.5%) | 0.25 (0.06–1.01) | 0.052 |
| >50 km | 29.4% (7.8–67.2%) | 34.5% (15.1–61.0%) | 1.26 (0.18–8.86) | 0.814 |

**Overall side-effects × distance interaction:** LRT χ²=8.94, df=2, **p=0.011.** Probabilities and contrasts were derived from the interaction model containing reported BPG side effects, distance to the follow-up facility, age, sex, duration of BPG prophylaxis and injection pain. Model-based probabilities were averaged over sex, prophylaxis duration and injection-pain categories, with continuous covariates evaluated according to the estimated marginal-means reference grid. Because the interaction analysis was exploratory and estimates in the more distant strata were imprecise, subgroup estimates should be interpreted cautiously.

**Supplementary Table S5**. Secondary-outcome sensitivity and model-diagnostic analyses

| Analysis | Estimate for ≥80% vs <80% BPG uptake | 95% CI | P value / diagnostic |
| --- | --- | --- | --- |
| Crude logistic regression | 0.20 | 0.12–0.33 | <0.001 |
| Adjusted for age and sex | 0.23 | 0.13–0.39 | <0.001 |
| Adjusted for age, sex and BPG prophylaxis duration | 0.25 | 0.14–0.44 | <0.001 |
| Influence sensitivity: excluding observation with highest Cook’s distance | 0.24 | 0.13–0.41 | <0.001 |
| Model discrimination | AUC 0.718 | 0.667–0.768 | DeLong 95% CI |
| Maximum Cook’s distance | 0.017 | — | 9 observations exceeded 4/n screening threshold |
| Separation assessment | No separation detected | — | Model estimates finite |
| Multicollinearity | No problematic multicollinearity | — | VIFs approximately 1.04–1.33 |
| Calibration | Predicted and observed probabilities showed reasonable agreement | — | Assessed graphically |

AUC, area under the receiver operating characteristic curve; BPG, benzathine penicillin G; CI, confidence interval; VIF, variance inflation factor. The secondary outcome was self-reported occurrence of one or more episodes of recurrent ARF during the preceding 12 months. The primary adjusted secondary-outcome model included age, sex and duration of BPG prophylaxis. The influence sensitivity analysis excluded the observation with the highest Cook’s distance.
