# Supplementary material for: Implications for sustaining secondary prophylaxis for rheumatic heart disease in Ethiopia: Uptake and determinants in a multicentre cross-sectional study
Source: PLoS Negl Trop Dis. 2026 Sep 11;20(9):e0014703. doi: 10.1371/journal.pntd.0014703 (PMC13588498; doi:10.1371/journal.pntd.0014703)
Supplement: Supplementary file 6 [file pntd.0014703.s007.docx]

**S6 Table. STROBE checklist for reporting the multicentre cross-sectional study**

*Determinants of uptake of benzathine penicillin G secondary prophylaxis for rheumatic heart disease in Ethiopia: A multicentre cross-sectional study*

This checklist is based on the STROBE recommendations for cross-sectional studies and has been adapted to map the reporting items to the manuscript.

| **Section** | **Item** | **STROBE reporting item (paraphrased)** | **Location in revised manuscript** | **How addressed** | **Final page/line** |
| --- | --- | --- | --- | --- | --- |
| Title and abstract | 1a | Identify the study design in the title or abstract. | Abstract / Methods | Reported as a multicentre facility-based cross-sectional study. | 2 |
|  | 1b | Provide an informative and balanced abstract summary. | Abstract | Background, methods, principal results and cautious conclusions reported. | 2 |
| Introduction | 2 | Explain the scientific background and rationale. | Introduction | Burden of RHD, role of BPG secondary prophylaxis, and evidence gap in Ethiopia described. | 3 |
|  | 3 | State specific objectives and any prespecified hypotheses. | Introduction, final paragraph | Objectives include BPG uptake, associated factors, and association with reported recurrent ARF manifestations. | 4 |
| Methods | 4 | Present key elements of the study design early. | Methods – Study design/setting | Multicentre facility-based cross-sectional design specified. | 5 |
|  | 5 | Describe setting, locations and relevant dates. | Methods – Study setting | Three referral hospitals in Ethiopia and November 2023–November 2024 study period reported. | 5 |
|  | 6 | Give eligibility criteria and sources/methods of participant selection. | Methods – Study participants | Echocardiography-confirmed RHD, ≥12 months of prescribed IM BPG prophylaxis, clinic attendance; eligible attendees consecutively approached. | 5 |
|  | 7 | Define outcomes, exposures, predictors, confounders and effect modifiers; give diagnostic criteria where applicable. | Methods – Variables / Statistical analysis | Primary outcome defined as ≥80% prescribed BPG uptake; secondary outcome defined as reported recurrent ARF manifestations; exposures and adjustment strategy described. | 6 |
|  | 8 | Describe data sources and measurement methods for variables of interest. | Methods – Data collection | Semi-structured questionnaire, trained data collectors, medical-record review, echocardiographic and clinical information described. | 6 |
|  | 9 | Describe efforts to address potential sources of bias. | Methods – Study participants/Data collection; Discussion – Limitations | Consecutive recruitment, standardized questionnaire administration, chart verification and limitations concerning clinic-based selection/reporting described. | 5-18 |
|  | 10 | Explain how study size was determined. | Methods – Sample size | Sample-size calculation/target should be reported here; verify final manuscript wording. | 7 |
|  | 11 | Explain handling of quantitative variables and any categorisation. | Methods – Statistical analysis | Age modelled continuously per 10-year increase; linearity assessed against natural spline; category boundaries reported for grouped exposures. | 7 |
|  | 12a | Describe statistical methods, including control of confounding. | Methods – Statistical analysis; S1 Table | Binary logistic regression with exposure-specific, a priori adjustment sets; ORs/aORs and 95% CIs reported. | 7 |
|  | 12b | Describe methods used to examine subgroups and interactions. | Methods – Statistical analysis; S4 Table | Exploratory BPG side-effects × distance interaction evaluated using an interaction model and LRT. | 7 |
|  | 12c | Explain how missing data were addressed. | Methods – Statistical analysis; S2/S3 Tables | Final analytical variables had no missing observations among n=400; screening-stage incomplete records described separately. | 7 |
|  | 12d | Describe methods accounting for sampling strategy, if applicable. | Methods – Study participants | Consecutive recruitment used; no probability-sampling weights applied. | 5 |
|  | 12e | Describe sensitivity analyses. | Methods – Statistical analysis; S2, S3 and S5 Tables | Influence analyses, additional adjustment, conservative duplicate-profile sensitivity analysis, and secondary-outcome sensitivity analyses reported. | 7 |
| Results | 13a | Report numbers of individuals at each stage. | Results – Participant flow | 553 screened; 153 not enrolled; 400 enrolled and analysed. | 8 |
|  | 13b | Give reasons for non-participation at each stage. | Results – Participant flow | 61 ineligible, 72 declined, 20 had insufficient clinical records | 8 |
|  | 13c | Consider use of a flow diagram. | Results / explained in words only | Explained in words only and believed to be sufficient for such a cross-sectional study | NA |
|  | 14a | Give participant characteristics and information on exposures/confounders. | Results; Table 1 | Demographic, socioeconomic, geographic, clinical and treatment-related characteristics reported. | 9 |
|  | 14b | Indicate numbers with missing data for variables of interest. | Results; S2 Table | No missing observations in final analytical variables (0/400). | Suppl |
|  | 15 | Report outcome-event numbers or summary measures. | Results | 305/400 (76.3%) achieved ≥80% BPG uptake; reported recurrent ARF manifestations summarized by uptake group. | 9 |
|  | 16a | Give unadjusted and adjusted estimates with precision; state adjustment variables and rationale. | Results; Table 2; S1 Table | Crude and adjusted ORs with 95% CIs reported; detailed exposure-specific adjustment sets provided in S1 Table. | 10 |
|  | 16b | Report category boundaries when continuous variables are categorized. | Methods; Tables 1–2 | Distance, duration, income, waiting time, transport cost and other grouped variables reported with explicit boundaries. | 9-12 |
|  | 16c | Translate relative estimates into absolute risk where relevant. | Not applicable / secondary outcome descriptive proportions | Cross-sectional odds ratios are primary effect measures; observed outcome proportions are also presented where informative. | NA |
|  | 17 | Report other analyses, including interactions and sensitivity analyses. | Results; S2–S5 Tables; S1–S2 Figs | Sensitivity analyses, exploratory interaction, diagnostics and calibration analyses reported. | Suppl |
| Discussion | 18 | Summarize key results with reference to objectives. | Discussion, opening paragraph | Principal uptake estimate and major associated factors summarized. | 13 |
|  | 19 | Discuss study limitations, including direction/magnitude of potential bias or imprecision. | Discussion – Limitations | Cross-sectional design, clinic-based recruitment, screening exclusions, self/reported recurrent ARF manifestations, residual confounding and other limitations discussed. | 17-18 |
|  | 20 | Give a cautious overall interpretation considering objectives, limitations, multiplicity and other evidence. | Discussion / Conclusion | Associations interpreted non-causally and in relation to Ethiopian and international evidence. | 13-18 |
|  | 21 | Discuss generalisability of the study results. | Discussion – Limitations/Generalisability | Findings framed for people with RHD attending referral follow-up services in Ethiopia; limits to broader generalisation acknowledged. | 13-18 |
| Other information | 22 | Give the source of funding and role of funders. | Funding statement | We don’t receive any specific funding for this research | NA |

Note: STROBE = Strengthening the Reporting of Observational Studies in Epidemiology; BPG = benzathine penicillin G; RHD = rheumatic heart disease; ARF = acute rheumatic fever; OR = odds ratio; aOR = adjusted odds ratio; CI = confidence interval; LRT = likelihood-ratio test. This study-specific checklist is a reporting aid; the official STROBE checklist remains the authoritative source.
