# Supplementary material for: Implications for sustaining secondary prophylaxis for rheumatic heart disease in Ethiopia: Uptake and determinants in a multicentre cross-sectional study
Source: PLoS Negl Trop Dis. 2026 Sep 11;20(9):e0014703. doi: 10.1371/journal.pntd.0014703 (PMC13588498; doi:10.1371/journal.pntd.0014703)
Supplement: Supplementary file 7 [file pntd.0014703.s008.docx]

**S1 Fig. Calibration plot for the adjusted distance–BPG uptake model.**

Calibration of the logistic regression model examining distance to the RHD follow-up facility and high benzathine penicillin G uptake. Observed proportions of participants achieving ≥80% BPG uptake are plotted against mean model-predicted probabilities across groups of predicted risk. The dashed 45° line represents perfect agreement between observed and predicted probabilities.
