# Supplementary material for: Implications for sustaining secondary prophylaxis for rheumatic heart disease in Ethiopia: Uptake and determinants in a multicentre cross-sectional study
Source: PLoS Negl Trop Dis. 2026 Sep 11;20(9):e0014703. doi: 10.1371/journal.pntd.0014703 (PMC13588498; doi:10.1371/journal.pntd.0014703)
Supplement: Supplementary file 8 [file pntd.0014703.s009.docx]

S2 Fig. Calibration plot for the secondary-outcome model of reported recurrent ARF manifestations.

Observed proportions of participants reporting carditis during the preceding 12 months are plotted against mean model-predicted probabilities across groups of predicted probability. The dashed 45° line represents perfect agreement between observed and predicted probabilities.
